# Derivation of the System Equation for Null-Balanced Total-Power Radiometer System NCS1

**DOI:** 10.6028/jres.099.006

**Published:** 1994

**Authors:** Sunchana P. Pucic

**Affiliations:** National Institute of Standards and Technology, Boulder, CO 80303-3328

**Keywords:** broadband, calibration, noise power, noise temperature, null-balanced, system equation, total power radiometer section

## Abstract

A system equation of a recently developed null-balanced, total-power radiometer system is rigorously derived. Delivered noise power and temperature is related to available power (temperature) through an extension of the mismatch factor to broadband systems. The available power ratio *α*_g_, the available gain *G_g_* and the delivered power ratio (efficiency) *η*_1_ are defined. Properties of idealized, but in principle realizable components such as an infinitely directive isolator and a lossless matched waveguide-below-cutoff attenuator are used. A cascading technique is repeatedly applied to the fundamental noise equation. Mathematically modeling the experimental procedure of sequentially attaching the two noise standards and the unknown source to the system input, we obtain the system of three equations that can be solved for the noise temperature of the unknown noise source.

## 1. Introduction

The system equation of a recently developed Noise Calibration System Model 1 (NCS1) is rigorously derived in this article. The NCS1, recently built at the National Institute of Standards and Technology, is used to measure thermal noise from coaxial noise sources. It consists of two noise standards and a null-balanced, total-power radiometer. The radiometer downconverts the amplified RF noise signal to a 30 MHz IF frequency. A precision waveguide-below-cutoff (WBCO) attenuator is used to achieve a balanced operations mode. An unbased square law diode serves as a detector. Nulling and resolution control are implemented at the post-detection, DC stage. The system is described in more detail in the accompanying articles [1] and [2].

The noise power available from a noise source and measured by the NCS1 is calculated according to the system equation. This article presents a rigorous derivation of that equation. Initially, the equation is derived for an idealized, simplified system (the simplified equation). Modifications of the simplified system needed to actually implement the NCS1, and the resulting modifications of the simplified system equation, are described later. Deviations from ideal behavior are treated in [2].

## 2. Derivation of the Simplified System Equation

### 2.1 Basic Considerations

Several topics are briefly reviewed, in preparation for a derivation of the system equation.

#### 2.1.1 Symbols

In the following text, terms a noise source and a noise generator are used interchangeably; the subscript g designates a general, unspecific origin of the noise signal (e.g., *P*_g_). The specific noise sources are identified with subscripts s for a noise standard (e.g., *P*_s_) and *x* for the unknown (e.g., *P_x_*). Terms the unknown noise source and the DUT (the device under test) are used interchangeably. The subscript a stands for the ambient temperature (as in *P*_a_).

Capital letter subscripts designate parts of the radiometer. The capital A stands for the attenuator, *G* for the amplifier, I for the isolator, and SW for the input switch.

For reflection coefficients within the radiometer, *Γ* signifies a reflection coefficient looking backward, in the direction opposite to the net power flow.

The subscript *i* in many of the quantities signifies that quantity's dependency on the existing input conditions. Initially, *i* stands for 1, 2, or *x*, that is, the conditions of having the noise standard 1, the noise standard 2, or the unknown noise source *x* attached to the input. Later on, *i* stands for s, a, *or x*, that is, the conditions of having the nonambient noise standard, the ambient noise standard, or the unknown noise source *x* attached to the input.

#### 2.1.2 The Measurement Quantity

The quantity measured by the NCS1 is the electromagnetic noise power *P*, in watts; however, it is the noise temperature *T*, in kelvins (K), that is customarily reported. Neglecting quantum effects, the two quantities are proportional to each other according to the well known relationship

P=kBT, (1)

where *k* is the Boltzmann constant = 1.38·10^−23^ J/K, and *B* is the system noise bandwidth in hertz.

The quantities *P* and *T* are the *available* noise power and the *available* temperature: the power or temperature that would be delivered by a source to a load, such as a power meter, under the ideal conditions of a complex conjugate match.

In contrast, the *delivered* power *P*_del_ (or temperature *T*_del_) is the power (temperature) that is actually delivered to a (generally mismatched) load.

#### 2.1.3 The Mismatch Factor

The available and the delivered powers (temperatures) are related by

$$P_{\mathrm{del}}=MP,$$ (2)

and

$$T_{\mathrm{del}}=MT,$$ (3)

where *M* is the mismatch factor, a real number between 0 (total mismatch), and 1 (perfect match). The mismatch factor is a function of the reflection coefficients of the source and the load. Power delivered to a load from sources having the same available power, but different reflection coefficients, is therefore variable and dependent on the input conditions.

In broadband systems of bandwidth *B*, the mismatch factor, if used as in Eqs. (2) and (3) is strictly defined as

$$M\int_{B}M(f)\mathrm{gd}f,$$ (4)

where *g* is the transfer function of the radiometer, and *f* is the frequency.

In principle, the mismatch factor can be evaluated according to Eq. (4). However, as long as the elements constituting *M* do not change appreciably across the measurement band, the use of a constant mismatch factor evaluated conveniently at mid-band produces negligible errors. Proper engineering design must assure a compliance with Eq. (4) and the error analysis must evaluate a deviation from the ideal conditions [3,4].

#### 2.1.4 Passive Two-Ports

If a noise generator with the available noise temperature *T*_g_ is connected to the input of a *passive*, linear two-port held at the temperature *T*, the available temperature *T*_out_ at the output of the two-port can be calculated from fundamental thermodynamic principles. It is given by [5, 3] by

$$T_{\text{out}}=T_{g}\alpha_{g}+T(1-\alpha_{g}).$$ (5)

The quantity *α*_g_ is a ratio of the available power at the output of the two-port to the available power at the input of the two-port: *α*_g_ *= P*_out_*/P*_in_. The quantity *α*_g_ is an *in situ* quantity; it depends not only on the parameters of the two-port, but also on those of the two-port's environment [5], Specifically, it depends on the reflection coefficient looking into the network preceding the two-port, that is, the (possibly equivalent in a Thevenin sense) generator, This dependence on the input conditions is indicated by the subscript g. The quantity *α*_g_ characterizes the lossiness of the two-port and has a value between 0 (an infinitely lossy two-port) and 1 (a lossless two-port). A full definition of *α*_g_, applied to the specific passive two-port within its environment, is given in Sec. 2.3.

The first term *T*_g_ *α*_g_ in Eq. (5) describes how the input noise signal is modified by a passage through the two-port. The second term *T*(1−*α*_g_) describes the noise contribution of the two-port itself.

#### 2.1.5 Active Two-Ports

If a noise source with the available noise temperature *T*_g_ is connected to the input of an *active* linear two-port such as an amplifier, the available temperature *T*_out_ at the output of the two-port is given by

$$T_{\text{out}}=G_{g}(T_{g}+T_{\mathrm{eg}}).$$ (6)

The quantity *G_g_* is the available gain of the active two-port. Similar to *α*_g_, it is a ratio of the available power at the output of the two-port to the available power at the input of the two-port, *G*_g_*≡P*_out_/*P*_in_. As an *in situ* quantity, it is dependent on the input conditions, as emphasized by the subscript g. A full definition, applied to the specific active two-port within its environment, is given in Sec. 2.3.

The quantity *T*_eg_ in Eq. (6) accounts for the noise contribution of the active two-port, a function of the circuit design and only marginally dependent on the physical temperature. The noise that is generated by the active two-port and is available at its output, is referred to its input as the *effective input noise temperature T*_eg._ The subscript g emphasizes its dependency on the reflective properties of the (possibly equivalent) generator (Sec. 2.3).

#### 2.1.6 A Cascade

From the definitions of *α* and *G* it follows that for a cascaded chain of *n* elements, the overall *α*_casc_ *= α*_1,_*α*_2_ *… a_n_*, and *G*_casc_ *= G*_1_ *G*_2_*…G_n_*.

### 2.2 Basic Assumptions

The derivation of the system equation is based on repeated applications of Eqs. (5) and (6) to the elements of the cascaded chain of the radiometer. The conditions assumed to hold are:

- Steady state
- Single-mode propagation in the transmission lines
- Lossless transmission lines and ideal connectors
- Linear elements throughout, except for the square-law power detector
- Broadband components across the bandwidth *B*
- Thermal noise (i.e. noise having a Gaussian amplitude distribution and a flat power spectrum across the noise bandwidth *B*) generated by the noise sources
- All passive components in the radiometer at the ambient temperature *T*_a_.
- A linear WBCO attenuator in its dial settings.

### 2.3 The Simplified Radiometer System

**Fig. 1:**
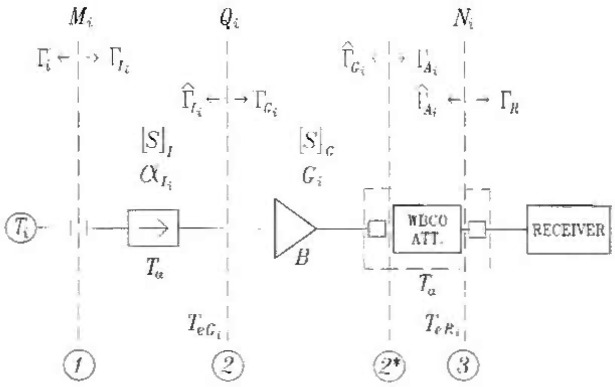
Simplified system block diagram.

For the purpose of deriving the system equation, the radiometer is reduced to essential components: an isolator, an RF amplifier, a WBCO attenuator with its matching pads (Sec. 2.3.2 and [6]), and a receiver containing a square law detector (Fig. 1). The lossiness of the input section of the radiometer is combined with the lossiness of the isolator. The isolator itself is initially treated as a simple lossy, reciprocal two-port, and later (Sec. 2.3.2) it assumes the nonreciprocal properties. The amplifier is the element assumed to define the system bandwidth.

The two noise standards arc characterized by their available noise temperatures *T*_1_ and *T*_2_ and their reflection coefficients *Γ*_1_, and *Γ*_2_. The DUT has a known (measured) reflection coefficient *Γ*_x_ Its available temperature is the quantity under test.

The radiometer input signal is the broadband noise generated by the three noise sources: the two noise standards and the DUT. The three sources are sequentially attached to the radiometer input port and the noise power is adjusted by the attenuator so that the receiver measures the same power in all three cases.

The radiometer reflection coefficient (at the input plane 1 in Fig. 1) is $\Gamma_{l_{i}}$ In general, it varies with the attenuator setting, since it depends on the (variable) $\Gamma_{A_{j}}$, according to [7]:

$$\Gamma_{1_{i}}=S_{11_{j}}+\frac{S_{12_{j}}S_{21_{j}}\Gamma_{G_{j}}}{1-S_{22_{i}}\Gamma_{G_{j}}},$$ (7)

while the term $\Gamma_{G_{j}}$, is, in turn, given by

$$\Gamma_{G_{i}}=S_{11_{G}}+\frac{S_{12_{G}}S_{21_{G}}\Gamma_{{Α}_{i}}}{1-S_{22_{G}}\Gamma_{{Α}_{i}}}.$$ (8)

In the preceding two equations, the *S*-parameters [*S*]_1_ characterize the isolator, while [*S*]_G_ pertain to a cascade of the amplifier and the left-sided matching pad of the WBCO attenuator.

The effective input noise temperature of the amplifier *T_eGj_*, varies with the input conditions. The effective input noise temperature of the receiver *T*_eRi_, varies with both the input conditions and the attenuator settings.

#### 2.3.1 Equations Modeling the Measurement Procedure

The measurement procedure consists of attaching the two noise standards and the DUT to the radiometer input port in sequence, and adjusting the attenuator settings until a balanced condition is achieved.

With the first noise standard (with available noise power *kBT*_1_ and reflection coefficient *Γ*_1_) attached to the radiometer input, the receiver detects a certain amount of delivered power *P*_del_:

$$\begin{array}{l} kB\{[T_{1}\alpha_{I_{1}}+T_{a}(1-\alpha_{I_{1}})+T_{{\mathrm{eG}}_{1}}]G_{1}\cdot \alpha_{A_{t}}+T_{a}(1-\alpha_{A_{1}})+\\ T_{{\mathrm{eR}}_{t}}\}\cdot N_{1}=P_{\mathrm{del}}. \end{array}$$ (9)

The term *G*_1_ (and likewise *G*_2_ and *G*_x_ in the following equations) refers to the available gain of the amplifier/left-matching-pad cascade (Sec. 2.1.6).

As the second noise standard (with available noise power *kBT*_2_ and reflection coefficient *Γ*_2_) is attached to the radiometer input, the attenuator is adjusted so that the receiver detects *the same* delivered power *P*_del_ as previously:

$$\begin{array}{l} kB\{[T_{2}\alpha_{l2}+T_{a}(1-\alpha_{l2})+T_{{\mathrm{eG}}_{2}}]G_{2}\cdot \alpha_{A2}+T_{a}(1-\alpha_{A2})+\\ T_{{\mathrm{eR}}_{2}}\}\cdot N_{2}=P_{\mathrm{del}}. \end{array}$$ (10)

Finally, as the DUT (with available noise power *kBT_x_* and reflection coefficient *Γ*_x_) is attached to the radiometer input, the attenuator is again adjusted so that the receiver detects the same delivered power *p*_del_ as in the previous two cases:

$$\begin{array}{l} kB\{[T_{x}\alpha_{lx}+T_{a}(1-\alpha_{lx})+T_{{\mathrm{eG}}_{x}}]G_{x}\cdot \alpha_{Ax}+\\ T_{a}(1-\alpha_{Ax})+T_{{\mathrm{eR}}_{x}}\}\cdot N_{x}=P_{\mathrm{dels}} \end{array}$$ (11)

where *k* is the Boltzmann constant, *B* is the limiting system bandwidth, *T_i_* (*i* =1,2,*x*) is the available noise temperature of the noise sources attached to the input, and *α*_1;_ and $\alpha_{A_{i}}$ are the available power ratios (Sec 2.1), *in situ* quantities. They are defined for the specific passive two-ports *within* their respective environments [5], as

$$\alpha_{l_{i}}=\frac{(1-{\left|\Gamma_{i}\right|}^{2})\cdot |S_{21_{l}}{|}^{2}}{(1-|\overset{⌢}{\Gamma_{l_{i}}}l^{2})11-S_{11_{i}}\Gamma_{i}l^{2}}$$ (12)

for the isolator, and

$$\alpha_{A_{i}}=\frac{(1-|{\overset{⌢}{\Gamma }}_{G_{i}}{|}^{2}\cdot |S_{21_{A_{i}}}{|}^{2}}{(1-|{\overset{⌢}{\Gamma }}_{A_{i}}{|}^{2})|I-S_{11}{\;}_{{\;}_{A_{i}}}{\overset{⌢}{\Gamma }}_{G_{i}}{|}^{2}}$$ (13)

for the attenuator.

In Eqs. (12) and (13) $S_{21_{l}},S_{11_{l}}\;\;\;\;\text{and}\;\;\;\;S_{21_{A_{i}}},S_{11_{A_{i}}}$ are the *S*-parameters of the isolator and the attenuator. The *S*-parameters of the attenuator vary with the attenuator setting.

The terms $\Gamma_{i},{\overset{⌢}{\Gamma }}_{I_{i}},{\overset{⌢}{\Gamma }}_{G_{i}},\;\;\;\text{and}\;\;\;{\overset{⌢}{\Gamma }}_{A_{i}}$, in the same equations are the reflection coefficients shown in Fig. 1. The fact that $\alpha_{l_{i}}$ and $\alpha_{A_{i}}$ have reflection coefficients looking either directly into the sources (*Γ_i_*), or indirectly into the sources through one or more two-ports ( ${\overset{⌢}{\Gamma }}_{I_{i}},{\overset{⌢}{\Gamma }}_{G_{i}},\;\;\;\text{or}\;\;\;{\overset{⌢}{\Gamma }}_{A_{i}}$), explicitly shows their dependency on the input conditions.

$T_{{\mathrm{eG}}_{i}}$ and $T_{{\mathrm{eR}}_{i}}$, are the effective input noise temperatures, in kelvins, of the amplifier and the receiver. The effective input noise temperature $T_{{\mathrm{eG}}_{i}}$ of the RF amplifier depends on the input conditions, i.e., on the reflection coefficients of the three noise sources *Γ_i_.* The effective input noise temperature of the receiver $T_{{\mathrm{eR}}_{i}}$ depends on the reflection coefficients of the three noise sources, as well as on the reflection coefficient looking backward into the attenuator at its three different settings ${\overset{⌢}{\Gamma }}_{A_{i}}$.

The available gain of the amplifier/left-matching-pad combination *G_i_*, *in situ* quantity, is given by

$$G_{i}=\frac{(1-|{\overset{⌢}{\Gamma }}_{l_{i}}{|}^{2}\cdot |S_{21_{G}}{|}^{2}}{\left(1-|{\overset{⌢}{\Gamma }}_{G_{i}}{|}^{2}\right)|1-S_{11_{G}}{\overset{⌢}{\Gamma }}_{l_{i}}{|}^{2}}$$ (14)

The terms ${\overset{⌢}{\Gamma }}_{l_{i}}$ and ${\overset{⌢}{\Gamma }}_{G_{i}}$ in Eq. (14) explicitly show the dependency of the available gain *G_i_* on the input conditions. In the case of ${\overset{⌢}{\Gamma }}_{l_{i}}$, this dependency is direct:

$${\overset{⌢}{\Gamma }}_{l_{i}}=S_{22_{l}}+\frac{S_{12_{l}}S_{21_{l}}\Gamma_{l}}{1-S_{11}\Gamma_{l}}.$$ (15)

In the case of ${\overset{⌢}{\Gamma }}_{G_{i}}$, the dependency is indirect, through the ${\overset{⌢}{\Gamma }}_{l_{i}}$, dependency on the input conditions:

$${\overset{⌢}{\Gamma }}_{G_{i}}=S_{22_{G}}+\frac{S_{12_{G}}S_{21_{G}}{\overset{⌢}{\Gamma }}_{l_{i}}}{1-S_{11_{G}}{\overset{⌢}{\Gamma }}_{l_{i}}}.$$ (16)

A mismatch factor *N_i_* is defined at the plane 3 in Fig. 1 as

$$N_{i}=\frac{\left(1-|{\overset{⌢}{\Gamma }}_{{Α}_{i}}{|}^{2})(1-|\Gamma_{R}{|}^{2}\right)}{|1-{\overset{⌢}{\Gamma }}_{{Α}_{i}}\Gamma_{R}{|}^{2}},$$ (17)

where *Γ*_R_ is the reflection coefficient looking into the receiver, and ${\overset{⌢}{\Gamma }}_{{Α}_{i}}$ is the reflection coefficient-looking backward into the attenuator, defined as

$${\overset{⌢}{\Gamma }}_{{Α}_{i}}=S_{22_{A_{i}}}+\frac{S_{12_{A_{i}}}S_{21_{A_{i}}}{\overset{⌢}{\Gamma }}_{G_{i}}}{1-S_{11_{A_{i}}}{\overset{⌢}{\Gamma }}_{G_{i}}}.$$ (18)

#### 2.3.2 Idealized Elements Assumptions

Equations (9)–(11) simplify considerably under the following assumptions:

- Infinitely directive ( $S_{12_{f}}=0$), but not necessarily lossless or matched isolator
- Large gain preceding the attenuator
- Lossless WBCO attenuator ( $\alpha_{A_{i}}=1$)

The first assumption results in the following simplifications:

$$\begin{array}{l} \Gamma_{l_{i}}=\Gamma_{l}(=S_{11_{l}});\;\;\;\;\;{\overset{⌢}{\Gamma }}_{I_{i}}={\overset{⌢}{\Gamma }}_{I}(=S_{22_{l}});\;\;\;\;\mathrm{and}\;\;\;{\overset{⌢}{\Gamma }}_{G_{i}}=\\ {\overset{⌢}{\Gamma }}_{G}(=S_{22_{G}})\\ G_{i}=G\\ T_{{\mathrm{cG}}_{i}}=T_{{\mathrm{eG}}_{i}};\;T_{{\mathrm{cR}}_{i}}\neq f\{\Gamma_{i}\}. \end{array}$$

Because of the second assumption, the temperature of the input noise signal, once it reaches the input of the receiver, is much higher than the receiver effective input noise temperature $T_{{\mathrm{eR}}_{i}}$. Consequently, the *variability* of the receiver effective input noise temperature, due to changes in the reflection coefficient of the receiver equivalent source ${\overset{⌢}{\Gamma }}_{A_{i}}$ (which in turn are related to different attenuator settings), becomes negligible: $T_{{\mathrm{eR}}_{i}}$ becomes the constant *T*_cR_.

The implications of the third assumption are discussed next. An ideal WBCO attenuator is inherently a lossless device, because the attenuation is achieved by the input signal propagating under the below-cutoff conditions. The input signal is therefore partially reflected and partially transmitted; no component of the input signal is absorbed, so there are no losses. (Small losses within the imperfect metal walls of the implemented attenuator can be neglected in this derivation.) The term $\alpha_{A_{i}}$ becomes 1, and consequently its noise contribution $T_{a}(1-\alpha_{A_{i}})$ vanishes.

The concept of attenuation is valid only in a non-reflecting environment [7]. The WBCO attenuator has a pair of (lossy) matching pads that are placed at the input and output ports to fulfill the requirement. The two reference planes (planes 2* and 3 in Fig. 1) are positioned between the WBCO attenuator proper and the matching pads, assuring a good bilateral match, while maintaining the lossless character of the WBCO attenuator proper.

The mismatch factor *N_i_* depends on *Γ*_R_ and ${\overset{⌢}{\Gamma }}_{A_{i}}$ [Eq. (17)]. The attenuator padding assures $\Gamma_{R}={\overset{⌢}{\Gamma }}_{G}\approx 0$. The condition *Γ*_r_ = 0 reduces the expression for the mismatch factor *N_i_* to $\left(1-|{\overset{⌢}{\Gamma }}_{A_{i}}{|}^{2}\right)$. Under the assumption that *Γ*_G_=0, the expression for ${\overset{⌢}{\Gamma }}_{A_{i}}$ in turn, reduces to $S_{22_{A_{i}}}$. The WBCO attenuator, postulated to be a lossless device, must satisfy the Iosslessness condition $\left|S_{22_{A_{i}}}{|}^{2}=(1-|S_{21_{A_{i}}}{|}^{2}\right)$ [7], Under the assumptions that the attenuator is lossless and matched, the expression for the mismatch factor *N_i_* is drastically simplified to $N_{i}=|S_{21_{A_{i}}}{|}^{2}$.

With all of the above simplifications in mind, the initial set of three Eqs. (9)–(11) now become

$$kB\{[T_{1}\alpha_{l_{1}}+T_{a}(1-\alpha_{l_{1}})+T_{\mathrm{eG}}]G+T_{\mathrm{cR}}\}|S_{21_{A_{1}}}{|}^{2}=P_{\mathrm{del}},$$ (19)

$$kB\{[T_{2}\alpha_{l_{2}}+T_{a}(1-\alpha_{l_{2}})+T_{\mathrm{eG}}]G+T_{\mathrm{cR}}\}|S_{21_{A_{2}}}{|}^{2}=P_{\mathrm{del}},$$ (20)

$$kB\{[T_{x}\alpha_{l_{x}}+T_{a}(1-\alpha_{l_{x}})+T_{\mathrm{cG}}]G+T_{\mathrm{cR}}\}|S_{21_{Ax}}{|}^{2}=P_{\mathrm{del}},$$ (21)

